# High-Performance Macroporous Free-Standing Microbial Fuel Cell Anode Derived from Grape for Efficient Power Generation and Brewery Wastewater Treatment

**DOI:** 10.3390/molecules29122936

**Published:** 2024-06-20

**Authors:** Jin-Zhi Sun, Quan-Cheng Shu, Hong-Wei Sun, Yu-Can Liu, Xiao-Yong Yang, Yan-Xiang Zhang, Gang Wang

**Affiliations:** 1 Yantai Engineering & Technology College, Yantai 264006, China; 2 School of Environmental and Material Engineering, Yantai University, Yantai 264005, China; 3 School of Civil Engineering, Yantai University, Yantai 264005, China

**Keywords:** microbial fuel cells, carbonized grape, extracellular electron transfer, brewery wastewater, 16S rRNA

## Abstract

Microbial fuel cells (MFCs) have the potential to directly convert the chemical energy in organic matter into electrical energy, making them a promising technology for achieving sustainable energy production alongside wastewater treatment. However, the low extracellular electron transfer (EET) rates and limited bacteria loading capacity of MFCs anode materials present challenges in achieving high power output. In this study, three-dimensionally heteroatom-doped carbonized grape (CG) monoliths with a macroporous structure were successfully fabricated using a facile and low-cost route and employed as independent anodes in MFCs for treating brewery wastewater. The CG obtained at 900 °C (CG-900) exhibited excellent biocompatibility. When integrated into MFCs, these units initiated electricity generation a mere 1.8 days after inoculation and swiftly reached a peak output voltage of 658 mV, demonstrating an exceptional areal power density of 3.71 W m^−2^. The porous structure of the CG-900 anode facilitated efficient ion transport and microbial community succession, ensuring sustained operational excellence. Remarkably, even when nutrition was interrupted for 30 days, the voltage swiftly returned to its original level. Moreover, the CG-900 anode exhibited a superior capacity for accommodating electricigens, boasting a notably higher abundance of *Geobacter* spp. (87.1%) compared to carbon cloth (CC, 63.0%). Most notably, when treating brewery wastewater, the CG-900 anode achieved a maximum power density of 3.52 W m^−2^, accompanied by remarkable treatment efficiency, with a COD removal rate of 85.5%. This study provides a facile and low-cost synthesis technique for fabricating high-performance MFC anodes for use in microbial energy harvesting.

## 1. Introduction

The rapid growth of the global economy and population has spurred the extensive exploitation and utilization of traditional fossil energy sources [1]. However, the non-renewable nature of fossil fuels, coupled with increasing demand and consumption, has led to their gradual deletion [2]. Consequently, the disparity between energy supply and demand has become increasingly pronounced. Furthermore, the world annually generates vast quantities of sewage. In 2022 alone, China’s total sewage discharge reached 63.9 billion tons, with approximately 26.0 million tons of chemical oxygen demand (COD) discharged in sewage [3]. Sewage is abundant in organic matter, rendering it a significant potential energy source. Theoretically, the complete oxidation of organic matter in sewage could yield approximately 1.16 kW·h·m^−3^ of energy [4]. However, traditional sewage treatment methods demand substantial energy input and fail to facilitate the recycling of the rich organic content present in sewage. In order to solve the two serious problems of energy shortages and water environment pollution, research into wastewater utilization technologies is urgently needed, which is also a sustainable development direction for sewage treatment practice in the future.

Microbial fuel cells (MFCs) represent a recent development in the intersection of environmental and energy fields, enabling simultaneous sewage treatment and the recovery of energy from organic matter [5]. As a multi-disciplinary energy production technology, MFCs offer innovative solutions for addressing water pollution and energy shortages [6]. As a promising green and sustainable technology, MFCs can use electrochemically active biofilms as anode catalysts to directly convert the chemical energy contained within wastewater into green electricity, without generating additional pollution [7]. This characteristic presents broad application prospects in wastewater treatment and energy recovery. Moreover, MFCs exhibit reduced susceptibility to temperature and environmental variations compared to other technologies. Additionally, the technology produces minimal sludge output, alleviating concerns regarding subsequent sludge treatment. Due to its advantages of cleanliness, efficiency, safety, pollution-free operation, mild operating conditions, noise-free operation, and modularity, MFC technology has received extensive attention and research in recent years [1,8]. Therefore, employing MFCs as platforms and utilizing microorganisms with extracellular electron transfer (EET) capabilities enables the simultaneous degradation of pollutant and recovery of energy. This dual functionality holds profound significance in mitigating the energy crisis and addressing environmental pollution challenges.

Current research on MFCs is primarily focused on several key aspects: (i) improving the performance of MFCs, with a particular emphasis on preserving original performance levels in scaled-up systems [9]; (ii) investigating the mechanisms of EET and direct interspecies electron transfer (DIET) in microorganisms [10]; (iii) exploring the commercial applications of MFCs [11]. Despite significant advancements in MFC output power and efficiency, the main challenge in practical applications remains the comparatively low power density when compared to other types of electrochemical devices. In principle, several factors can affect the output power density of MFCs, including bacteria loading capacity, electron transfer rate, circuit resistance, the degradation rate of organic substrate, and more [12,13,14,15]. Anode materials play an important role as sites for microbial attachment and electron transfer, thus significantly impacting the performance of MFCs across all aspects [16]. From the perspective of material properties, numerous factors influence the microbial adhesion and biological electron transfer processes in MFCs. These include electrochemical activity, hydrophilicity, specific surface area, surface functional groups, and various physicochemical and interfacial properties [17,18]. Furthermore, MFCs’ anode materials should possess characteristics such as stability, durability, affordability, and accessibility [19,20]. Therefore, the search for more efficient and cost-effective anode materials is an important direction for the development of MFC technology.

In the early stages, anode materials for MFCs were often drawn from other battery electrode materials. Among these, traditional carbon materials emerged as popular choices due to their affordability and stability. However, they possess drawbacks such as high electron transfer resistance, limited specific surface area, and poor biocompatibility, which hinders bacterial attachment and biofilm formation, thereby limiting MFC performance. To overcome these limitations and develop high-performance, long-term stable anode materials for MFCs, researchers have synthesized a range of functional nanomaterials. These include carbon-based nanomaterials like graphene [21] and carbon nanotubes [22], various conductive polymers [23], and metal nanoparticles [24], which serve as either anode materials or modifiers for MFCs. While materials like graphene, carbon nanotubes, and various nanomaterials have demonstrated enhanced MFC performance, they also exhibit drawbacks such as poor mechanical strength, low electrochemical stability, and limited biocompatibility, which hinder their practical application. In addition to these materials, carbon materials derived from high-temperature carbonized biomass are frequently utilized as MFC anodes [25,26]. Derived from natural sources, these materials are abundant in elemental contents. Furthermore, the rich heteroatom doping that occurs during carbonization confers excellent biocompatibility upon the carbonized material [26]. Additionally, the formation of groups such as pyrrole nitrogen and graphitic nitrogen during the carbonization process can accelerate electron transfer [25]. High-temperature carbonization also results in the creation of rich multi-level micro–nano channels, which serve as an excellent structural foundation for microbial growth and attachment, as well as the adsorption of mediators [27]. In addition, this type of carbonized natural biomass material is widely available and easy to prepare, endowing it with a distinct advantage as an anode material for MFCs.

Grape has been cultivated as a common industrial crop in China. The main content of grape is carbohydrate (more than 80%). There are also some other contents, like protein, dietary fiber, and metal elements. The organic waste of grape from thinning fruit accumulated on a plantation, in addition to occupying space, usually attracts insects and rodents. For these reasons, finding alternatives for their employment will be of great benefit. In this study, carbonized grape (CG) materials were successfully prepared and investigated as MFC anodes for brewery wastewater treatment. Firstly, the surface morphology, composition, structure, specific surface aera, and pore size of the CGs were characterized and analyzed. Subsequently, the electrochemical properties of the CGs, including the conductivities, diffusion properties, ion diffusion coefficients, and capacitive performance, were evaluated to analyze electrode reaction properties, processes, and mechanisms. Furthermore, the performance of CGs as MFC anodes was systematically evaluated, including their output voltage, polarization curve, power density, coulomb efficiency, and COD removal rate. Additionally, the bioelectrochemical activity and microbial community structure of the biofilm were investigated. Finally, the practical application performance of MFCs equipped with CG anodes was assessed using brewery wastewater.

## 2. Results and Discussion

### 2.1. Preparation and Characterization of CGs

**Figure 1:**
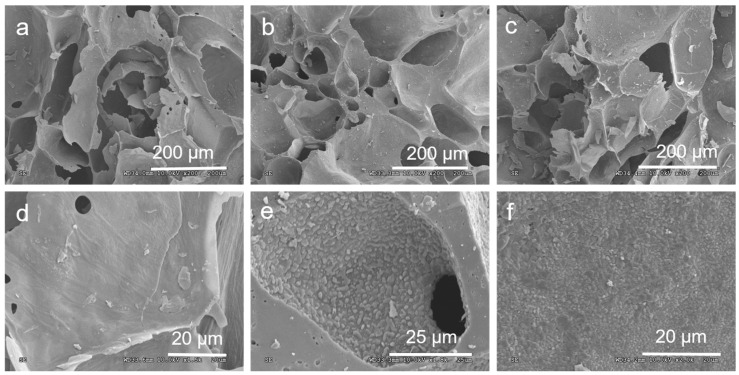
SEM images of (**a**) CG-800, (**b**) CG-900, and (**c**) CG-1000; and enlarged SEM images of (**d**) CG-800, (**e**) CG-900, and (**f**) CG-1000.

The SEM images of CG-800, CG-900, and CG-1000 clearly depicted macroporous structures with sizes ranging from tens to hundreds of interconnected macroporous channels (Figure 1a–c). Despite noticeable shrinkage post-carbonization, centimeter-sized porous monoliths were obtained. Differences in roughness were observed among the surface structures of CGs at different carbonization temperatures (Figure 1d–f). This discrepancy could be attributed to the varying consumption rates of carbon and non-carbon molecules from the carbon network during carbonization, resulting in the development of porous and rough surfaces. Specifically, the surface of CG-900 appeared rougher, with more pronounced protrusions. These structural characteristics are advantageous for bacterial attachment and may expedite the start-up time of MFCs. Firstly, the electron-accepting ability of the heteroatoms could create net positive charge on adjacent carbon atoms in the carbon materials. In addition, the heteroatoms could induce surface acidity and hydrophilicity to significantly enhance the bacteria sorption. The pore structure maximizes the contact area between the bacterial cell and the surface and affects bacterial biofilm formation. The large surface area and porous structures containing micro-, meso-, and macropores not only offer large accessible surfaces for bacterial colonization, but also provide more active sites for electron mediators to promote EET and shorten the start-up time of MFCs.

**Figure 2:**
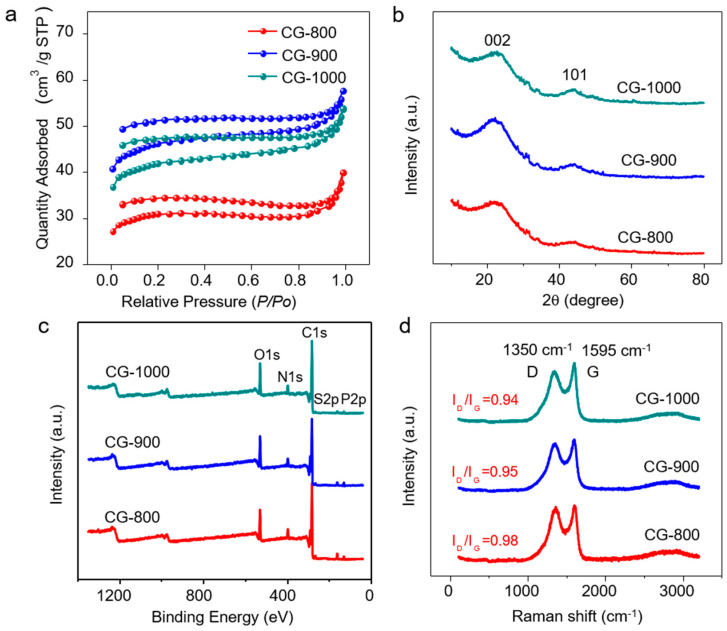
(**a**) Nitrogen adsorption–desorption isotherms; (**b**) XRD patterns; (**c**) XPS survey spectra; and (**d**) Raman spectra of CG−800, CG−900, and CG−1000.

The specific surface area and dV/dlog (D) pore volume curve of the prepared CGs were determined using the Brunauer–Emmett–Teller (BET) method (Figure 2a). The specific surface areas of CG-800, CG-900, and CG-1000 MFC anodes were 105 cm^2^ g^−1^, 157 cm^2^ g^−1^, and 147 cm^2^ g^−1^, respectively (Table S1). With an increase in carbonization temperature from 800 °C to 900 °C, the specific surface area increased by nearly 1.5 times, while it slightly decreased by 9.49% when the temperature further increased to 1000 °C. In addition, the different pore textures of the scaffolds also affected the specific surface area. As shown in Figure S1, the intensity of CG-900 was higher than the others, indicating the presence of a larger amount of pore textures (Table S1).

The small-angle XRD spectra (Figure 2b) of CG materials exhibited two major broad diffraction peaks at 2θ angles of 23° and 45° corresponding to the d-spacing of 0.39 nm and 0.22 nm, attributed to the (002) and (100) diffraction peaks of carbon [28]. The elemental composition was further examined by XPS, revealing the presence of C, O, N, S, and P, as depicted in Figure 2c and Table S2. The results indicate that the degree of carbonization and the carbon ratio increased with temperature. The high-resolution C1s spectra were then analyzed, and the predominant peaks were deconvoluted into four components: sp2 hybridized carbon C=C (284.7), sp3 hybridized state carbon C–C (285.5), C–O/C–N (286.4), C=O (288.3), and carbon oxides (283.94) (Figure S2). The CG-800, CG-900, and CG-1000 samples exhibited different N contents, approximately 3.22%, 1.08%, and 0.97%, respectively. Three different binding forms of nitrogen were identified, including pyridinic N at 398.3 eV, pyrrolic N at 400.3 eV, and graphitic N at 400.9 eV [29]. Additionally, trace amounts of P and S atoms were detected in the CGs materials. The XPS-S2p results revealed sulfur doping, indicated by the formation of –C–S–C– and –C–S(O)x–C– bonds within the carbon lattice, while the P2p peaks exhibited P–O and P–C bonding (Figure S3). These heteroatoms likely contribute to open-edge sites and improve mass transport within the porous structures. The Raman spectra of CGs exhibited broad bands at 1350 cm^−1^ and 1595 cm^−1^, corresponding to defects in the graphitic layers (D-band) and graphitic layers (G-band), respectively (Figure 2d). The presence of D-bands and G-bands was consistent with the model of vortex-layer disordered graphene sheets containing hard carbons. As the temperature increased, the intensity ratio of the D-band and G-band (ID/IG) decreased due to enhanced carbon graphitization, which was consistent with the XPS results [30,31,32]. In addition, the coherence length *La* based on Equation (1) [33,34,35] increased during the heat treatment from 18.1 nm (CG-800) to 18.3 nm (GTO-900) and 18.8 nm (CG-1000). These values indicate an increase in crystallinity along *ab* planes.

(1) $$\mathrm{La}\left(\mathrm{nm}\right)=\left(2.4\times 10^{-10}\right)\times \lambda_{\mathrm{nm}}^{4}\times \left(\frac{I_{G}}{I_{D}}\right)$$

### 2.2. Electrochemical Properties of CGs

**Figure 3:**
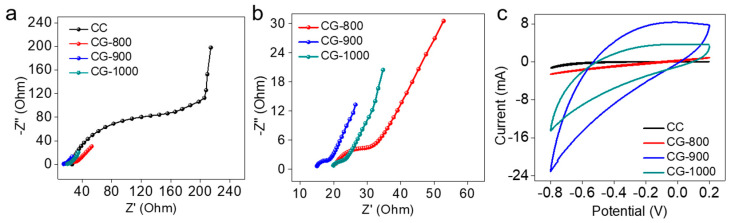
(**a**) EIS Nyquist plots; (**b**) enlarged view in the high-frequency range; and (**c**) CVs of CC, CG−800, CG−900, and CG−1000.

The conductivities and diffusion properties of the synthesized CG MFC anode materials were assessed using the EIS method conducted in 50 mM phosphate-buffered solution (PBS). The results were illustrated through Nyquist curves and further analyzed with equivalent circuits (Figure 3a,b). Each EIS curve exhibited a distinct semicircle in the high-frequency range followed by a linear trend in the low-frequency range. The diameter of the semicircle represented the charge transfer resistance (Rct) at the electrode/electrolyte interface. This parameter is indicative of the relative activity of different electrodes towards small redox shuttles within an electrolyte [36,37]. Compared to CC, the Rct values of CGs were significantly lower, with the order being CC (221 ohms) > CG-800 (13.3 ohms) > CG-1000 (4.72 ohms) > CG-900 (4.39 ohms), indicating a much faster electron transfer rate for the CG anodes.

In addition to Rct, ion diffusion resistance also plays a key role in causing power loss in MFCs. By utilizing specific equations, the ion diffusion coefficient (D) can be calculated from plots in the low-frequency region (Table S3). The D values for CG-800, CG-900, CG-1000, and CC were determined to be 4.56 × 10^−7^ cm^2^ s^−1^, 1.81 × 10^−6^ cm^2^ s^−1^, 1.23 × 10^−6^ cm^2^ s^−1^, and 1.53 × 10^−9^ cm^2^ s^−1^, respectively (Table S3). Compared to CC, the D values of CG materials increased by nearly 3 orders of magnitude. This significant enhancement suggests that ion diffusion readily occurs between the surface of the CG material and the anolyte, thereby facilitating accelerated the ion diffusion within the electrode.

The capacitive performance of the MFC anode is significantly influenced by its large specific surface area and porous structure. The integration of an internal capacitor into the anode has been shown to enhance transient charge storage behavior, thereby improving MFC performance [38,39]. As depicted in Figure 3c, CGs exhibit substantially higher capacitive current compared to CCs, owing to their increased number of active sites and higher specific surface area for biocatalytic redox reactions. This indicates that CGs can provide a larger active surface area for bacterial attachment [36,40]. Consequently, CGs represent promising anode materials for improving MFC performance, as revealed by both electrochemical analysis and ion diffusion coefficient measurements.

### 2.3. MFC Performance Based on the CGs

**Figure 4:**
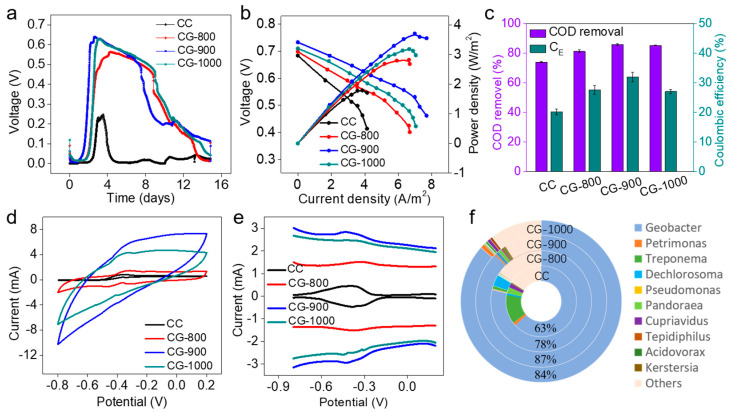
(**a**) Voltage output versus time of MFCs with various anodes during start−up time; (**b**) polarization and power density curves; (**c**) positive correlation between COD removal efficiency and columbic efficiency; (**d**) CVs of different anodes after acetate is depleted. Electrolyte: 50 mM PBS containing 2 g L^−1^ sodium acetate. Scan rate: 5 mV s^−1^; (**e**) DPVs of different anodes with biofilm; (**f**) structure of microbial community at different anodes.

The classic H-shaped MFC (HMFC) was utilized to study the performance of MFCs equipped with an independent CG anode and carbon fiber cathode in batch mode. Following inoculation with mixed bacteria for 1.8 days, the output voltage of the CG-900 anode exhibited a rapid increase, stabilizing at a maximum voltage of 0.64 V (Figure 4a). The start-up times for CG-1000 and CG-800 were 2.3 days and 2.5 days, respectively, with each reaching its maximum voltage of 0.63 V and 0.57 V, respectively. Nevertheless, the ordinary CC-based MFC achieved a relatively low initial maximum voltage of 0.31 V, which took at least 3.1 days to reach. Following multiple operations, the CG anodes continued to display stable and repeatable voltage output cycles. Specifically, the stable voltages recorded for CG-800, CG-900, and CG-1000 were 0.57 V, 0.66 V, and 0.63 V, respectively.

The polarization curve and power density of the MFC equipped with various anodes were examined by changing the external load resistance after 2-month runs. In comparison to the CC anode, MFCs configured with CG anodes exhibited a slower decline in output voltage as the external load decreased gradually (Figure 4b). This observation suggests that the internal resistance of CGs was significantly lower owing to their fast charge transfer rate and superior conductivity. The areal and volumetric power density rankings were as follows: CG-900 (3.71 W m^−2^ at 7.00 A m^−2^) > CG-1000 (3.18 W m^−2^ at 6.65 A m^−2^) > CG-800 (2.82 W m^−2^ at 6.64 A m^−2^) > CC (1.78 W m^−2^ at 3.86 A m^−2^). Notably, the CG-900 anode achieved the highest maximum power density of 3.705 W m^−2^. This highlights that the MFC configured with CG anodes surpassed that of the CC-based MFC, with the CG-900 surface proving particularly suitable for high-performance MFC applications.

The coulomb efficiency and COD removal rate were analyzed after a full cycle of steady current output. The CG anodes exhibited higher COD removal efficiency (81.3 ± 0.98% for CG-800, 86.0 ± 0.72% for CG-900, 85.5 ± 0.20% for CG-1000) compared to the CC anode (74.2 ± 0.47%) (Figure 4c). Similarly, the coulombic efficiency of CG-900 anodes followed the same trend. Notably, CG-900 exhibited the highest efficiency at 32.0%, indicating its superior ability to convert chemical energy in substrates into electrical energy [37]. Moreover, the COD removal rate showed a positive correlation with columbic efficiency.

Furthermore, the bioelectrochemical activity of the biofilm formed on the anode was studied using cyclic voltammetry (CV). After stable operation for 30 days, the CG anodes showed redox couples at −0.33 V and −0.40 V versus Ag/AgCl (Figure 4d). An electroactive biofilm on the CG-900 anode produced a catalytic current density of 5.66 mA cm^−2^ at the potential of −0.33 V, which was 6.36 times higher than that of the CC (0.89 mA cm^−2^), indicating its superior conductivity and capacitance. The maximum current densities of CG-1000 and CG-800 were 4.32 mA cm^−2^ and 1.52 mA cm^−2^, respectively. The electron transfer mechanism between the electrode surface and bacteria was further studied using the more sensitive technique of differential pulse voltammetry (DPV) to eliminate the interference of capacitive and background signals (Figure 4e). The results indicated that all electroactive anodes displayed a wide redox potential window ranging from −0.25 V to −0.55 V, consistent with the broad redox windows of outer-membrane c-type cytochromes (OMCs). While the DPV curves exhibited similarities, differences in current intensity were observed. Specifically, CG-900 displayed a higher catalytic current (2.87 mA) compared to CG-1000 (2.48 mA), CG-800 (1.54 mA), and CC (0.49 mA), indicating a higher number of electroactive species and enhanced catalytic activity. Notably, the biofilm on the CG-900 anode produced two redox peaks at −0.42 V and −0.43 V. Importantly, a rapid electron transfer process was evident in the biofilm on the CG-900 surface, as the distance between the anodic peak and cathodic peak was less than 20 mV.

The microbial community structure on the anodes was determined using 16S rRNA gene sequencing technology (Figure 4f). The CG-900 anode exhibited an optimal morphology structure for capturing more bacteria. The results revealed a diverse community consisting of both electrochemically active microorganisms and non-electrochemically producing microorganisms residing on the anode. Some of the identified genera included Geobacter, Petrimonas, Treponema, and Dechlorosoma, among others. Among these, Geobacter was identified as the dominant microorganism. The CG-900 anode captured a higher quantity of bacteria, with a Geobacter content as high as 87.1%, significantly surpassing CG-1000 (84.2%), CG-800 (78.2%), and CC (63.0%). Additionally, Petrimonas, Treponema, and Dechlorosoma were also present in varying proportions on different electrodes. These findings suggest that electrogenic bacteria and non-electrogenic bacteria cooperate to produce electricity within the biofilm. The CGs facilitated the enrichment of electrochemically active microorganisms, further confirming that the superior electricity production performance of CGs MFCs is primarily attributed to the abundant electricigens loaded on the anodes.

**Figure 5:**
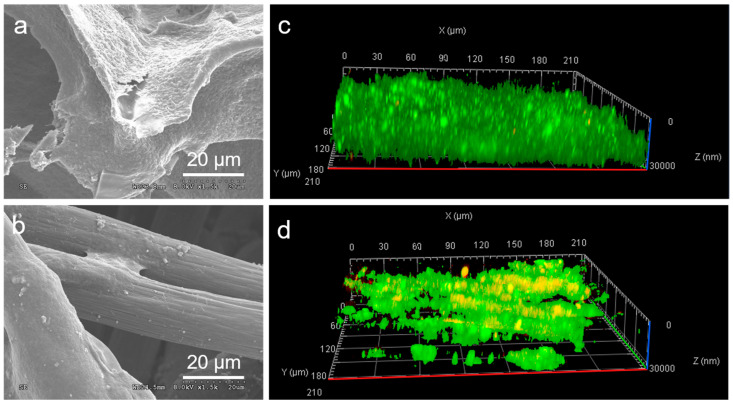
SEM images of biofilm growing on the surface of (**a**) CC and (**b**) CG−900; CLSM images of biofilm growing on the surface of (**c**) CC and (**d**) CG−900.

The morphology of biofilms formed on different anodes after 75 days of stable voltage output was further investigated (Figure 5). As depicted in Figure 5a, rod-shaped bacterial cells covered the entire surface and interior of the CG-900 electrode, forming a robust biofilm. These observations demonstrated that the open macroporous structure of CG-900 possesses a high bacterial loading capacity, ensuring adequate substrate transport to maintain internal colonization. Bacterial cells densely adhered to the surface of the CG-900 anode, indicating that its material surface demonstrated good interaction with microbial films. In contrast, biofilms formed only on the CC surface due to its low porosity and small specific surface area (Figure 5b). To validate this deduction, confocal laser scanning microscope (CLSM) images using an alive–dead stained assay were further studied to examine the distribution of biofilms. After 75 days of operation, it was observed that cell death on the CG-900 anode was negligible, indicating its good biocompatibility (Figure 5c). In contrast, a significant number of bacteria attached to the CC anode died, possibly due to its relatively low porosity, which limits substrate exchange and hampers the provision of internal bacterial biofilm growth (Figure 5d). This result indicates that the electrode surface of CG-900 was more conductive to the attachment of electroactive bacteria.

**Figure 6:**
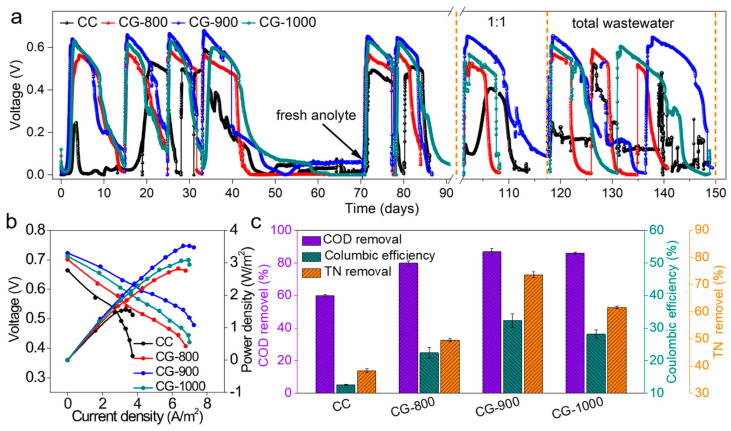
(**a**) Voltage output of MFCs with different anode materials in synthetic wastewater, brewery wastewater diluted with synthetic wastewater (1:1), and brewery wastewater; (**b**) polarization and areal power density curves of CG and CC anodes in brewery wastewater; (**c**) positive correlation between COD removal efficiency, columbic efficiency, and TN removal efficiency in brewery wastewater.

Furthermore, to evaluate its practical application feasibility, MFCs equipped with CGs anodes were tested using brewery wastewater. Brewery wastewater is characterized by a large volume of sewage discharge and high organic content (Table S5), making it suitable for biological treatment due to its non-toxic nature [41]. However, traditional treatment technologies entail high energy input and sludge treatment, significantly increasing system operating costs [42]. Moreover, MFCs have proven to be suitable for wastewater treatment, offering both pollutant removal and energy recovery benefits. The results showed that MFCs equipped with different anodes showed distinct responses to wastewater. As shown in Figure 6a, when the ratio of synthetic wastewater to brewery wastewater was 1:1, all three CG anodes consistently maintained regulated voltage output, with the MFC employing the CG-900 anode demonstrating rapid startup within a short timeframe. Furthermore, resilience after adversity was another important indicator for evaluating MFC performance. In this study, the stability and reliability of the MFC system were primarily assessed through continuous operation time. When the medium was refreshed after approximately 30 days without feeding, the voltage of the CGs recovered quickly without experiencing a significant drop. This observation suggests that the CG anode possesses better bacterial adhesion affinity and higher anode durability, which are advantageous for practical applications. When the anolyte was changed to pure beer wastewater, the voltage of the CG-900 anode MFC quickly rose to the maximum value of 0.65 V, and the power density reached 3.52 W m^−2^ (Figure 6b), with no significant decrease compared to the previous synthetic wastewater substrate. However, the MFC equipped with a CC anode could not recover its initial value in pure brewery wastewater substrate. The lower power density in this case could be attributed to various factors, such as the complex composition of the wastewater, deteriorating bacterial growth, low conductivity, and the presence of other electron acceptors. Despite the influence of these factors on the power generation performance of the MFCs, an areal power density of 3.52 W m^−2^ was still achieved by the CR-900 anode in brewery wastewater medium. Furthermore, CG-900 exhibited an excellent performance among reported anodes (Table S4).

The COD removal rates for CC, CG-800, CG-900, and CG-1000 in brewery wastewater were 59.8%, 79.8%, 86.8%, and 85.9%, respectively, while the columbic efficiencies were 12.6%, 22.4%, 32.3%, and 28.2%, respectively (Figure 6c). Simultaneously, the CG anodes also showed higher TN (total nitrogen) removal efficiencies, with TN removal rates of 38.3%, 49.5%, 73.6%, and 61.6%. Negassa reported that the simultaneous treatment of brewery wastewater and production of bioelectricity using double-chamber MFCs resulted in a COD removal efficiency of 83.0% [43]. Tessema studied the effect of electron acceptors in energy generation from brewery wastewater via a microbial fuel cell, indicating the COD removal rate and columbic efficiency were 64.3% and 25.0%, respectively [44]. Additionally, the COD removal rate, coulombic efficiency, and maximum power density exhibited a positive correlation in brewery wastewater substrates. These results verify that CG anodes could not only utilize acetate to generate electricity but also exhibit higher capabilities in actual brewery wastewater treatment.

## 3. Materials and Methods

### 3.1. Materials’ Synthesis and Anode Preparation

Shine Muscat grapes were sourced from a grape plantation in Yantai. Initially, grapes at the expanding stage with rounded shapes and identical weights (5 ± 0.01 g) were selected. These grapes were then washed with deionized water and subsequently dried in an electric thermostatic blast drying oven (DHG-9076A, Shanghai Jinghong Co., LTD, Shanghai, China). Following this, the dried grapes underwent calcination under nitrogen at temperatures of 800 °C, 900 °C, and 1000 °C with a heating rate of 5 °C min^−1^ and were maintained at each temperature for 2 h using an atmosphere furnace (GMF-17-205, Shanghai Dute Co., LTD, Shanghai, China). The porous carbon materials obtained from the aforementioned process were denoted as CG-800, CG-900, and CG-1000. Following this, the CG porous materials were trimmed to match the same weight of MFC anodes and tightly wrapped with titanium wire (99.9%) to facilitate connection to the external circuit for electricity generation data collection. The morphology, composition, and electrochemical properties of the CGs were analyzed using techniques such as X-ray diffraction (XRD), X-ray photoelectron spectroscopy (XPS), the determination of binding energies, Raman spectroscopy, Brunauer–Emmett–Teller (BET) analysis, electrochemical impedance spectroscopy (EIS), and cyclic voltammetry (CV) (Text S1).

The brewery wastewater was sourced from Qingdao Beer Co., LTD with a COD concentration of 1.84 g L^−1^. Additionally, a 0.05 M PBS solution was employed to dilute the obtained brewery wastewater, maintaining similar loading rates [45]. For the actual wastewater application test, the brewery wastewater was diluted with synthetic wastewater at a 1:1 ratio and injected into the MFC anode chamber, and this process was repeated for two cycles [46]. Following this, the practical application testing continued using raw brewery wastewater.

### 3.2. MFC Setup and Operation

The dual-chamber H-shaped MFC reactor comprised two equal-length rectangular glass bottles (100 mL each), operating in a batch feeding mode and separated by a cation exchange membrane. Carbon cloth (CC) was chosen as the control group for CGs, while the MFC cathode consisted of a graphite fiber brush. The anolyte composition consisted of PBS (50 × 10^−3^ M), sodium acetate (2 g L^−1^), vitamins (0.2 mg L^−1^), and minerals (82.1 mg L^−1^). The catholyte consisted of potassium ferricyanide and potassium chloride, both at concentrations of 50 × 10^−3^ M. A mixture of bacteria-rich MFC effluent and anaerobic sludge was used to inoculate the MFCs, which were then operated at 37 °C. An external resistor of 1 kΩ was connected to MFCs, while the cell voltage was collected and recorded by an online data collection system. A voltage exceeding 500 mV indicated successful MFC initiation. When the cell voltage value dropped to 100 mV, the electrode solution was replaced. The power generation performance of the MFCs was evaluated using power density and polarization curves (Text S2). Additionally, the COD removal rate, calculated by measuring the difference in COD between the inlet and outlet water, was used to evaluate the organic matter removal capacity of the MFCs (Text S2).

### 3.3. Biofilm Characterization

The morphology of the biofilm attached to the anodes was examined using a scanning electron microscope (SEM, Hitachi S-4300, Tokyo, Japan), while its viability was analyzed with a confocal laser scanning microscope (CLSM, inVia-Reflex, Chicago, IL, USA) (Text S3). Microbial community analysis was conducted after two months of steady and repeatable voltage output. DNA extraction was conducted following the operation manual of the DNA isolation kit (MP Biomedicals, FastDNA^TM^ Spin Kit for Soil, Solon, OH, USA), and it then was sequenced through the NovaSeq PE250 platform (Thermo, Waltham, MA, USA). The quality difference method was used to evaluate the biomass of different anodes by ultrasonic stripping biofilms (Text S4).

## 4. Conclusions

Three-dimensional carbon monolith MFC anodes with interconnected macroporous channels and rough surface structures were successfully fabricated using grape carbonization for brewery wastewater treatment. CG-900 showed superior electrochemical performance with an Rct of only 4.39 ohms. The enhanced electron transfer rate of the MFC was mainly from the enhanced directed electron transfer and also mediated electron transfer. In addition, CG anodes could enrich more electrochemically active microorganisms in anode biofilm. Overall, the CG-900 anode exhibited a maximum power density of 3.71 W m^−2^, which was 2.08-fold of CC. Despite being a relatively new technology, MFCs have the potential to play a role in solving global environmental problems in the future through continued research. Furthermore, the integration of conventional systems with MFCs can lead to economic feasibility and wider adoption of this technology.

## Data Availability

The original contributions presented in the study are included in the article/Supplementary Material; further inquiries can be directed to the corresponding author.

## Supplementary Materials

The following supporting information can be downloaded at https://www.mdpi.com/article/10.3390/molecules29122936/s1: Figure S1: The dV/dlog (D) pore volume curves of CG-800, CG-900, and CG-1000, Figure S2: High-resolution C1s XPS spectra of CG-800 (a), CG-900 (b), CG-1000 (c) and High resolution N1s XPS spectra of CG-800 (d), CG-900 (e), CG-1000 (f). The high-resolution C1s spectra of CGs contain C=C (284.7), C–C (285.5), C–O/C–N (286.4), C=O (288.3) and carbon oxides (283.94). The N1s peak arises mainly from pyridinic N (398.3 eV), pyrrolic N (400.3 eV), graphitic N (400.9 eV) respectively, Figure S3: High-resolution P2p XPS spectra of CG-800 (a), CG-900 (b), CG-1000 (c) and High resolution S2p XPS spectra of CG-800 (d), CG-900 (e), CG-1000 (f); Table S1: BET and pore size distribution of different samples, Table S2: The peak parameters of the different components, values given in percentage of total amount, Table S3: Fitted parameters of some elements in the equivalent circuit, Table S4: Comparison of MFC performances, Table S5: The brewery wastewater characteristics; Text S1: Characterization, Text S2: The analysis of polarization and power output curves and COD, Text S3: The morphology and viability of biofilms on different anodes, Text S4 The microbial community analysis. References [46,47,48,49,50,51,52,53,54,55,56,57,58,59] are cited in the supplementary materials.
