# Supplementary material for: High-Performance Macroporous Free-Standing Microbial Fuel Cell Anode Derived from Grape for Efficient Power Generation and Brewery Wastewater Treatment
Source: Molecules. 2024 Jun 20;29(12):2936. doi: 10.3390/molecules29122936 (PMC11206865; doi:10.3390/molecules29122936)
Supplement: Supplementary file 1 [file molecules-29-02936-s001.zip › molecules-3053850-Supporting Information.pdf]

Supporting Information

High-Performance Macroporous Free-Standing Microbial Fuel Cell Anode Derived from Grape for Efficient Power Generation and Brewery Wastewater Treatment

Jin-Zhi Sun [1], Quan-Cheng Shu [2], Hong-Wei Sun [2], Yu-Can Liu [3], Xiao-Yong Yang [2], Yan-Xiang Zhang [2] and Gang Wang [2,*]

[1] Yantai Engineering & Technology College, Yantai 264006, China
[2] School of Environmental and Material Engineering, Yantai University, Yantai 264005, China
[3] School of Civil Engineering, Yantai University, Yantai 264005, China
* Correspondence: gwang91@ytu.edu.cn

Supporting Information Contents:

Number of pages (including this page): 15

Number of Figures: 3

Number of Texts: 4

Number of Tables: 5

Text S1 Characterization

The X-Ray Diffraction (XRD) analysis was performed with Bruker D8 ADVANCE XRD spectrometer. The X-ray photoelectron spectroscopy (XPS) analysis was carried out on Thermo escalab 250Xi. Binding energies were calibrated using the adventitious carbon (C1s) = 284.5 eV. Raman spectra were collected using Renishaw inVia Reflex. The Brunauer-Emmett-Teller (BET) surface area was measured using the TriStar II 3020 instrument.

Electrochemical impedance spectroscopy (EIS) and cyclic voltammograms (CVs) experiments were performed on 1260 Impedance/gain-phase Analyzer (Solartron Metrology Inc., USA). Differential pulse voltammetry (DPV) was performed with a CHI 860D electrochemical workstation (Chenghua Inc., USA). All these tests were operated with a three-electrode chamber containing a MFC anode, a platinum-wire counter electrode, and an Ag/AgCl reference electrode with saturated KCl (potential vs the normal hydrogen electrode, NHE +198 mV). For EIS, the frequency range was from 100 kHz to 0.01 Hz with the direct current potential of 0.2 V. CV was recorded between -0.8 and 0.2 V. DPV was performed from -0.8 to 0.8 V with amplitude 60 mV, pulse width 200 ms and potential increment 6 mV.

Text S2 The analysis of polarization and power output curves and COD

The polarization and power output curves were obtained at the stable state of MFCs by varying the external resistance over a range from 40 Ω to 2 kΩ. The current and power density was normalized by the projected area or volume of the anode for analysis. Both current density and power density were normalized to the anode surface area. After an entire cycle, the influent and effluent from MFCs were immediately filtered through 0.22 mm pore-diameter cellulose acetate filters and then were analyzed for chemical oxygen demand (COD). The water sample (2 mL of influent or effluent) was added into a Test ‘N’ tube (Cat. 2125925-CN), heated by the DRB 200 thermostat and then analyzed by a DR 3900 VIS spectrophotometer following the manufacturer's instructions (Test ‘N’ tube, DRB 200 and DR3900, HACH Instruments, Inc. USA).

Text S3 The morphology and viability of biofilms on different anodes

The morphology of the electrode and biofilm attached to anodes was observed by Hitachi S-4300 scanning electron microscope (SEM). Bacteria immobilized on different anodes were fixed in 4% polyformaldehyde solution for 30 min at 4 °C, rinsed three times with PBS solution, and then dehydrated using graded ethanol solution (30,50, 70, 85, and 95% for one time, 100% for two times). After dried in air, samples were sputtered with gold.

The viability of bacteria in MFC anodes were analyzed with the LIVE/DEAD BacLight Bacterial. Viability Kit. Biofilms on different anodes were stained with two fluorescent dyes, SYTO-9 and PI kitaccording to the manufacturer's instruction. After staining, the biofilms were observed with the Leica TCS SP5 confocal laser scanning microscopy.

Text S4 The microbial community analysis

DNA extraction was conducted after two mouths steady and repeatable voltage output using Power Soil DNA Isolation Kit (MoBio Laboratories, Inc, Carlsbad, CA) following the manufacturer's instructions. DNA concentration was confirmed by a spectrophotometer (NanoDrop 2000c, Thermo, USA). High-throughput microbial community analysis was then conducted on the MiSeq platforms. Raw sequence data to NCBI Sequence Read Archive (SRA) was uploaded with accession number PRJNA45139. Universal primers 515F (5'-GTGCCAGCMGCCGCGGTTAA-3') and 907R (5'-CCGTCAATTCCTTTGAGTTT-3') were used for PCR amplifying V4 and V5 regions of the bacterial 16S rRNA gene. PCR product was mixed and purified with Qiagen Gel Extraction Kit (Qiagen, Germany). Sequencing libraries were generated using TruSeq DNA PCR-Free Sample Preparation Kit (Illumina, USA). Individual samples were barcoded in one run of an Illumina Hiseq platform (2500, Illumina, CA) to generate 250 bpparied-end sequencing reads. OTUs were generated by sequences (analyzed by Uparse software) with ≥97% similarity. Phylogenetic relationship was constructed by phylogenetically assigning sequences to achieve the phylum, order, class, family and genes level using the MOTHUR program with distance level of 0.03 and confidence threshold of 97% for the phylogenetic classification. Relative abundance of a certain sample was calculated by dividing its total sequences to the total sequences.

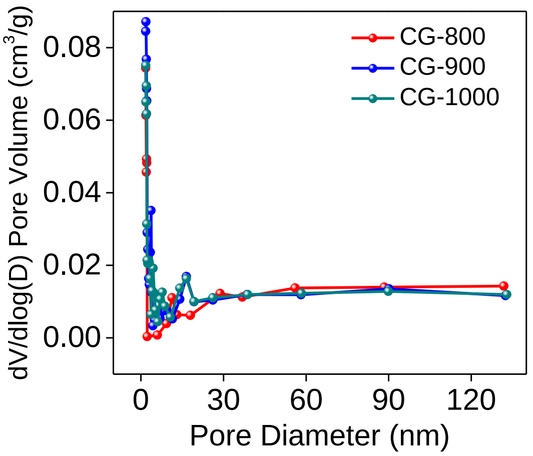

Figure S1. The dV/dlog (D) pore volume curves of CG-800, CG-900, and CG-1000.

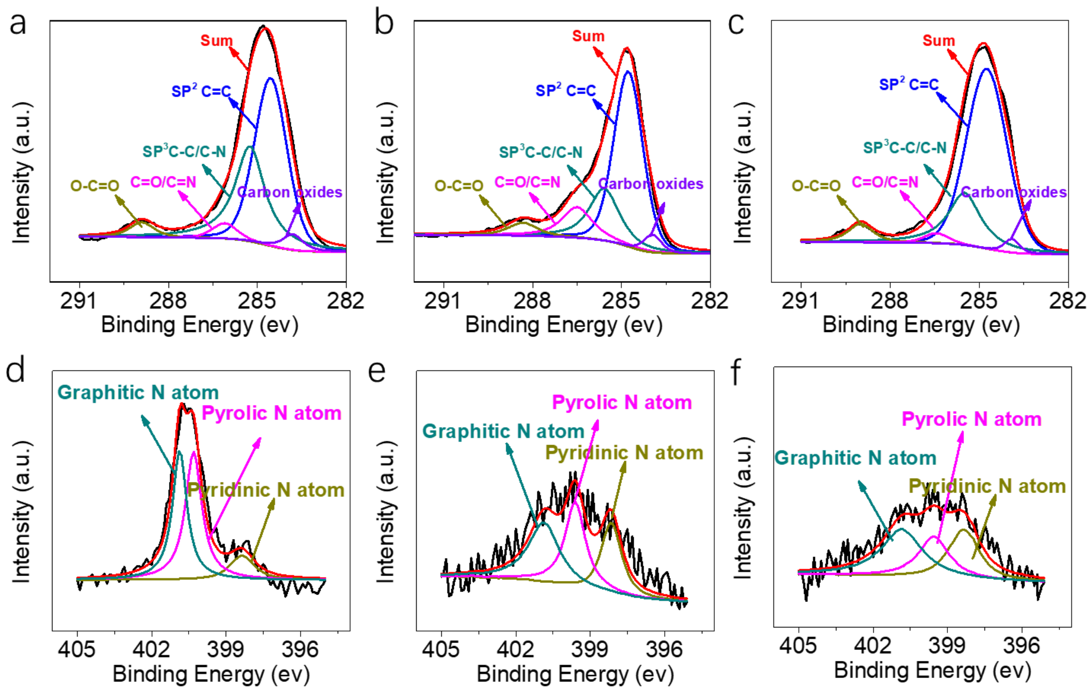

Figure S2. High-resolution C1s XPS spectra of CG-800 (a), CG-900 (b), CG-1000 (c) and High resolution N1s XPS spectra of CG-800 (d), CG-900 (e), CG-1000 (f).The high-resolution C1s spectra of CGs contain C=C (284.7), C-C (285.5), C-O/C-N (286.4), C=O (288.3) and carbon oxides (283.94). The N1s peak arises mainly from pyridinic N (398.3 eV), pyrrolic N (400.3 eV), graphitic N (400.9 eV) respectively.

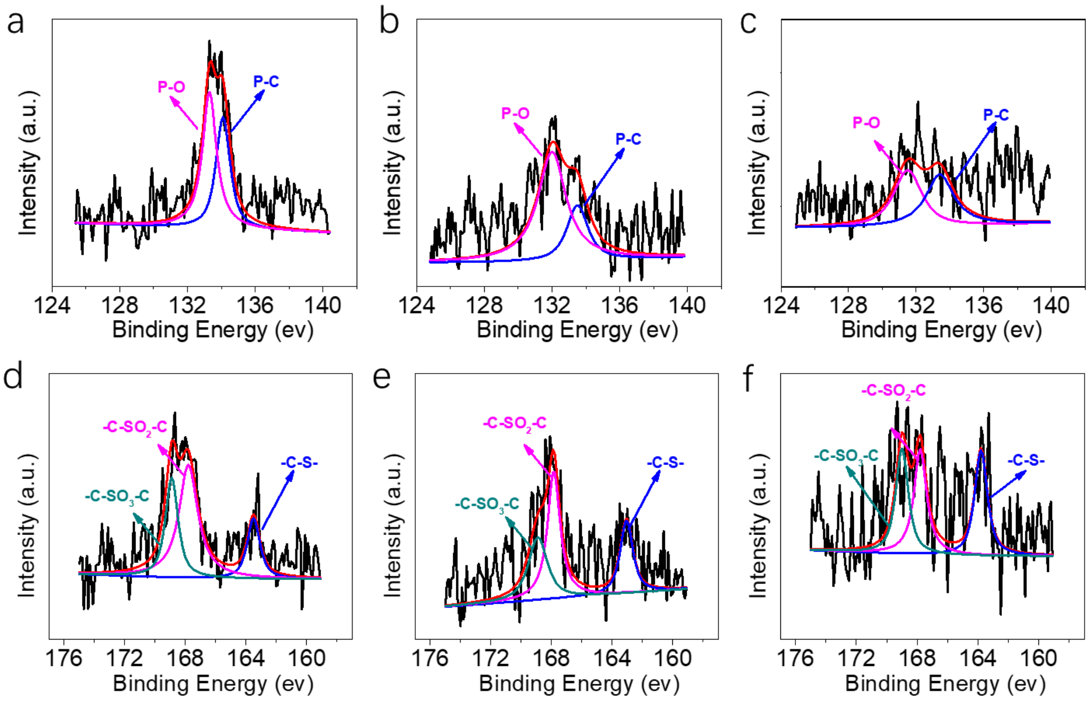

Figure S3. High-resolution P2p XPS spectra of CG-800 (a), CG-900 (b), CG-1000 (c) and High resolution S2p XPS spectra of CG-800 (d), CG-900 (e), CG-1000 (f).

Table S1. BET and pore size distribution of different samples

Samples	S_{BET} ($cm^2 \cdot g^{-1}$)	V_{total} ($cm^3 \cdot g^{-1}$)	$V_{meso/macro}$/ ($cm^3 \cdot g^{-1}$)	V_{micro} ($cm^3 \cdot g^{-1}$)
CG-800	104.82	0.0616	0.0207	0.0409
CG-900	156.76	0.0890	0.0304	0.0586
CG-1000	141.88	0.0831	0.0295	0.0536

Table S2. The peak parameters of the different components, values given in percentage of total amount.

Samples	C (at.%)	O (at.%)	N (at.%)	P (at.%)	S (at.%)
CG-800	78.7	16.34	3.22	1.11	0.63
CG-900	80.69	16.79	1.08	0.95	0.49
CG-1000	81.73	16.25	0.97	0.68	0.37

Table S3. Fitted parameters of some elements in the equivalent circuit.

Samples	Before enrichment of bacteria			After enrichment of bacteria	
	R_s	R_{ct}	D ($cm^2 \cdot s^{-1}$)	R_s	R_{ct}
Carbon cloth	26.36	221.43	1.53×10^{-9}	20.26	207.6
CG-800	19.84	13.25	4.56×10^{-7}	17.35	11.98
CG-900	14.65	4.39	1.81×10^{-6}	13.24	3.11
CG-1000	19.31	4.72	1.23×10^{-6}	18.61	3.61

The ion diffusion coefficient (D) can be calculated from the plots in the low-frequency region based on equations (S1) and (S2):

$$Z' = R_s + R_{ct} + \sigma_w \omega^{-0.5} \quad \text{(S1)}$$

$$D = \frac{R^2 T^2}{2S^2 F^4 \sigma_\omega^2 C^2 n^4} \quad \text{(S2)}$$

Where Z' is the real part of the impedance, ω is angular frequency, and σ_W represents the slope of Z' against $\omega^{-0.5}$. $R, T, n, F, S,$ and C are gas constant, absolute temperature, the mole of charge per ion, Faraday constant, surface area, and molar concentration of electrolyte ions, respectively. The D of CG-900 anode is estimated to be 1.81×10^{-6} $cm^2 \cdot s^{-1}$ about three magnitude that of CC electrode (1.53×10^{-9}), and also higher than that of other CG electrodes.

Table S4 Comparison of MFC performances.

Anode	Inoculum source	Substrate	P_{max}	Ref.
3D nitrogen-doped graphene aerogel	*S. oneidensis* MR-1	trypticase soy broth	1990 mW·m^{-2}	[S1]
Graphene-sponge composite	*S. oneidensis* MR-1	glucose	1570 mW·m^{-2}	[S2]
Nitrogen-doped Porous Carbon	mixed bacteria	acetate	2777.7 mW·m^{-2}	[S3]
three-dimensional polypyrrole network	mixed bacteria	glucose	3317 mW·m^{-2}	[S4]
carbonized phenolic foam	mixed bacteria	acetate	3980 mW·m^{-2}	[S5]
porous α-Fe_2O_3 nanofibers	mixed bacteria	acetate	1952 mW·m^{-2}	[S6]
graphite felt	mixed bacteria	brewery wastewater	305 mW·m^{2}	[S7]
graphite plate	mixed bacteria	brewery wastewater	80.01 mW·m^{-2}	[S8]
graphite granule	mixed bacteria	brewery wastewater	340 mW·m^{-3}	[S9]
graphite plate	mixed bacteria	brewery wastewater	173.1 mW·m^{-2}	[S10]
Carbon cloth	mixed bacteria	brewery wastewater	528 mW·m^{-2}	[S11]
graphite disc	mixed bacteria	brewery wastewater	330 mW·m^{-2}	[S12]
carbon cloth	mixed bacteria	brewery wastewater	483 mW·m^{-2}	[S13]
graphite felt	mixed bacteria	brewery wastewater	552 mW·m^{-2}	[S14]
CG-900	mixed bacteria	brewery wastewater	3520 mW·m^{-2}	This work

[S1] Yang Y, Liu T, Zhu X, et al. Boosting Power Density of Microbial Fuel Cells with 3D Nitrogen-Doped Graphene Aerogel Electrode [J]. Advanced Science, 2016, 3(8):

[S2] Xie X, Yu G, Liu N, et al. Graphene-sponges as high-performance low-cost anodes for microbial fuel cells [J]. Energy & Environmental Science, 2012, 5(5): 6862-6866.

[S3] Bi L, Ci S, Cai P, et al. One-step pyrolysis route to three dimensional nitrogen-doped porous carbon as anode materials for microbial fuel cells [J]. Applied Surface Science, 2018, 427: 10-16.

[S4] Li F, Wang D, Liu Q, et al. The construction of rod-like polypyrrole network on hard magnetic porous textile anodes for microbial fuel cells with ultra-high output power density [J]. Journal of Power Sources, 2019, 412: 514-519.

[S5] Zhu Y, Feng Y, Zhang L, et al. Economic affordable carbonized phenolic foam anode with controlled structure for microbial fuel cells [J]. Science of the Total Environment, 2022, 810:

[S6] Liu Y, Zhang X, Li H, et al. Porous α- α-Fe_2O_3 nanofiber combined with carbon nanotube as anode to enhance the bioelectricity generation for microbial fuel cell [J]. Electrochimica Acta, 2021, 391:

[S7] Miran W, Nawaz M, Kadam A, et al. Microbial community structure in a dual chamber microbial fuel cell fed with brewery waste for azo dye degradation and electricity generation [J]. Environmental Science and Pollution Research, 2015, 22(17): 13477-13485.

[S8] Cetinkaya A Y, Koroglu E O, Demir N M, et al. Electricity production by a microbial fuel cell fueled by brewery wastewater and the factors in its membrane deterioration [J]. Chinese Journal of Catalysis, 2015, 36(7): 1068-1076.

[S9] Angosto J M, Fernandez-Lopez J A and Godinez C. Brewery and liquid manure wastewaters as potential feedstocks for microbial fuel cells: a performance study [J]. Environmental Technology, 2015, 36(1): 68-78.

[S10] Zhang X, Zhu F, Chen L, et al. Removal of ammonia nitrogen from wastewater using an

aerobic cathode microbial fuel cell [J]. Bioresource Technology, 2013, 146: 161-168.

[S11] Feng Y, Wang X, Logan B E, et al. Brewery wastewater treatment using air-cathode microbial fuel cells [J]. Applied Microbiology and Biotechnology, 2008, 78(5): 873-880.

[S12] Katuri K P and Scott K. Electricity Generation From the Treatment of Wastewater With a Hybrid Up-Flow Microbial Fuel Cell [J]. Biotechnology and Bioengineering, 2010, 107(1): 52-58.

[S13] Wang X, Feng Y J and Lee H. Electricity production from beer brewery wastewater using single chamber microbial fuel cell [J]. Water Science and Technology, 2008, 57(7): 1117-1121.

[S14] Yu J, Park Y, Kim B, et al. Power densities and microbial communities of brewery wastewater-fed microbial fuel cells according to the initial substrates [J]. Bioprocess and Biosystems Engineering, 2015, 38(1): 85-92.

Table S5 The brewery wastewater characteristics

Brewery wastewater parameter	pH	COD (mg/L)	TOC (mg/L)	TN (mg/L)
Average ± SD	9.8 ± 0.1	1458 ± 14	401.5 ± 5.5	21.2 ± 0.3
